# A Specialized Pipeline for Efficient and Reliable 3D Semantic Model Reconstruction of Buildings from Indoor Point Clouds

**DOI:** 10.3390/jimaging10100261

**Published:** 2024-10-19

**Authors:** Cedrique Fotsing, Willy Carlos Tchuitcheu, Lemopi Isidore Besong, Douglas William Cunningham, Christophe Bobda

**Affiliations:** 1 Department of Graphic Systems, Institute for Computer Science, Brandenburg University of Technology Cottbus-Senftenberg, Platz der Deutschen Einheit 1, 03046 Cottbus, Germany; cunningh@b-tu.de; 2 Department of Mathematics and Data Science, Faculty of Sciences and Bio-Engineering Sciences, Vrije Universiteit Brussel, 1050 Brussels, Belgium; willy.carlos.tchuitcheu@vub.be; 3 Institute of Metallurgy, Clausthal University of Technology, 38678 Clausthal-Zellerfeld, Germany; besong.besong.lemopi.isidore@tu-clausthal.de; 4 Department of Electrical and Computer Engineering, University of Florida, 36A Larsen Hall, Gainesville, FL 116200, USA; cbobda@ece.ufl.edu

**Keywords:** point cloud, segmentation, building information modeling, 3D semantic model, IFC

## Abstract

Recent advances in laser scanning systems have enabled the acquisition of 3D point cloud representations of scenes, revolutionizing the fields of Architecture, Engineering, and Construction (AEC). This paper presents a novel pipeline for the automatic generation of 3D semantic models of multi-level buildings from indoor point clouds. The architectural components are extracted hierarchically. After segmenting the point clouds into potential building floors, a wall detection process is performed on each floor segment. Then, room, ground, and ceiling extraction are conducted using the walls 2D constellation obtained from the projection of the walls onto the ground plan. The identification of the openings in the walls is performed using a deep learning-based classifier that separates doors and windows from non-consistent holes. Based on the geometric and semantic information from previously detected elements, the final model is generated in IFC format. The effectiveness and reliability of the proposed pipeline are demonstrated through extensive experiments and visual inspections. The results reveal high precision and recall values in the extraction of architectural elements, ensuring the fidelity of the generated models. In addition, the pipeline’s efficiency and accuracy offer valuable contributions to future advancements in point cloud processing.

## 1. Introduction

Recent advances in laser scanning systems have facilitated the acquisition of 3D point cloud representations of scenes. Many high-precision and less expensive technologies for accurately scanning real-world indoor and outdoor scenes have been developed [1]. A direct consequence of this technological advance is the interest of actors in the fields of Architecture, Engineering, and Construction (AEC) in generating 3D models of existing buildings from high-precision scans. However, the manual reconstruction process has proven to be time-consuming, and even when performed by the same person, the results from the same point cloud are often non-uniform [2,3]. In view of these limitations, it is obvious that automation of the reconstruction process is necessary.

Automatic generation of 3D models of buildings from point clouds is a very active research branch in computer vision and computer graphics that finds applications in AEC domains (design, modelling, and planning), gaming, augmented reality, indoor navigation, etc. [4,5]. Generally known as building information models (BIMs), 3D semantic models of existing structures are increasingly generated automatically from high-precision cloud points [6]. In the construction industry, for example, such generated models can help in minimizing misunderstandings among different teams involved in the project, which results in a reduction of errors and construction costs [7].

Generating 3D semantical models of buildings from point clouds requires reliable processes for the identification of structural elements (rooms, walls, floors, ceilings, openings, etc.) and their interconnections. The unorganized nature of some point clouds of buildings and the presence of outliers such as furniture constitute a challenge during the extraction or identification of structural components. Despite the significant interest and multiple research works related to the automatic generation of 3D models of buildings from point clouds, the quality of the resulting models usually fails to accurately represent the scanned structures [8]. While some proposed solutions focus only on surface reconstruction without considering the interconnectivity between structures [9,10], other solutions use unreliable component detection methods (RANSAC, Hough transform, etc.) to identify components [11]. In addition, most proposed methods are limited to the reconstruction of single-story buildings [12].

We propose a new pipeline for 3D semantic model reconstruction of multi-level buildings using indoor point clouds. The proposed pipeline takes indoor point clouds of buildings as input. Without pre-processing (sampling, noise removal, etc.) operations on the dataset, architectural elements are extracted using special methods. Then, based on the relations between the extracted elements, a 3D semantic model of the building is generated. Unlike existing methods that proceed by global segmentation of point clouds, followed by the classification of the different blocks belonging to particular classes of structural elements (wall, floor, ceiling, etc.), we propose specialized detection methods for each class. By decomposing the extraction process into specific tasks, it becomes possible to focus on individual aspects of the structural components. This allows for the application of specialized algorithms adapted to perform each task, leading to more precise classification and the extraction of relevant features from the point cloud.

This research is the continuation of previous works. Hence, the different methods used for extracting the architectural components are either improvements of prior proposed methods or novel methods. The segmentation of point clouds into levels is performed using a point histogram distribution. The wall structures are extracted using 2D grid structures [13]. After extracting the wall structures, openings are identified via an occupancy 2D grid using a training classifier. From the 2D wall constellation, we propose new methods for room detection and ground extraction. Finally, the complete 3D semantic model is generated in Industry Foundation Classes (IFC) format using the hierarchical relationships between the detected components.

The paper is organized into five sections. Some relevant works related to the automatic generation of 3D models of buildings from point clouds are reviewed in Section 2. The steps of the proposed pipeline are described in Section 3. To demonstrate the reliability of the proposed method, we present the results of the execution of the pipeline on a set of indoor point clouds in Section 4, followed by conclusions in Section 5.

## 2. Related Works

The BIM standard has been a major revolution in the field of AEC in recent decades. The standardization and wide adoption of BIM concepts by AEC professionals in recent years confirm this trend [14]. Beyond a simple visualization tool, BIM is a working method in which the visual components of buildings are identified and described semantically (cost, material, lifespan, etc.). Such information can be used by stakeholders involved in projects carried out on an infrastructure during its life-cycle from planning to exploitation and maintenance. The non-existence of semantic models for some existing buildings is usually a hindrance to the use of BIM methods during maintenance or renovation. As a result of advances in high-precision laser scanning, many techniques have been proposed for the rapid reconstruction of 3D semantic models of existing buildings from scans [15,16]. A critical step common to 3D model reconstruction methods from point clouds is labelling architectural components observed in scans. The main differences between the proposed methods lie in the approaches used to extract structural elements and the processes used to identify links between structures in the final model. In this section, we review works related to the generation of semantic models of buildings from indoor scans.

To address the inefficiencies of manually generating 3D models of buildings from point clouds, Hong et al. [3] developed a semi-automatic process. After extracting horizontal planar structures, a filtering step was performed to eliminate noises like furniture. The rest of the point cloud was then projected onto a 2D plane to extract the floor boundary and wall segments. Since the generation of the BIM model was done manually in AutoCAD Revit 2013, they were able to easily represent equipment previously classified as clutters. Budroni and Boehm [17] proposed to extract planar surfaces from the point clouds using an iterative vertical and horizontal sweeping process. Following the direction of the real-world coordinate system, the horizontal planes were classified as either ceiling or floor, and the vertical planes were considered walls. The floor plan was extracted from the orientation of the walls previously detected, and using information from the walls (position and size) and floor plane, they successfully generated a 3D model. After aligning point clouds to a common reference space, Mura et al. [18] derived a graph-based representation that encodes the adjacency relations between previously detected planar components. They extracted permanent architectural components by using graph structures. The 3D representations of the permanent components were used to build a complex that divides the scene into polyhedral cells. The final model was built as a block of individual rooms by applying a multi-label optimization to the set of polyhedral cells. Tran et al. [19] proposed a shape grammar approach for generating navigable spaces and building elements in a hierarchical structure compatible with BIM standards. Using the shape grammar, they generated a 3D parametric model by repeatedly placing cuboids into spaces enclosed by points. After classifying the cuboids into building elements and spaces, the spaces were sequentially merged to form the final navigable and non-navigable spaces. Ultimately, it allowed the reconstruction of 3D models of complex environments from simple cuboid shapes. Topological relations between cuboid shapes, including adjacency, connectivity, and containment, were reconstructed in the final model. The placement, classification, merging processes, and topology reconstruction were governed by the grammar rules.

Considering that the architectural components are represented as planar surfaces, some authors proposed using traditional planar cluster segmentation methods such as RANSAC and Hough transforms. Jung et al. [2] suggested automatically detecting 3D geometric drawings of indoor structures using a RANSAC-based segmentation method, then manually building the model in a BIM software environment. To reconstruct a 3D model of a room from indoor point clouds, Xiong et al. [6] presented a method that first identified wall blocks with a Hough transform. The detection of openings was conducted on the detected segments by representing walls as voxels. Voxel cells were then classified as occupied, empty, or occluded using information from the room scans. After filling the occluded cells, they used features from a Support Vector Machine (SVM) classifier to extract doors and windows. Sanchez and Zakhor [20] employed model-fitting (RANSAC) on pre-segmented planar regions to represent basic architectural structures (ceilings, floors, walls, and staircases) while supposing that building interiors can be modelled as a set of planar primitives. The 3D model was generated by assembling the detected structures. Assuming the input point cloud is aligned with the XYZ axes, Oesau et al. [21] extracted permanent structures by partitioning the bounding box into volumetric cells. Horizontal structures (ceilings, floors, etc.) were detected using a vertical point distribution diagram. After detecting ceilings and floors, the input point cloud was segmented into horizontal slices representing the levels of the building. At each level, vertical structures were detected by fitting multi-scale feature-preserving lines and clustering in a global Hough transform space. Then volumetric cells from the previous segmentation were classified as empty or solid through an energy minimization problem solved by graph-cut. The 3D model was extracted as the union of all empty cells. In high-resolution point clouds, Díaz-Vilariño et al. [22] first detected permanent structures like walls with a region-growing segmentation method. Then, using a model-fitting (generalized Hough transform), they identified closed openings in each segment. Finally, a visual labelling process was carried out to reconstruct the 3D model. Thomson and Boehm [23] and Danielle Kwadjo [11] proceeded by segmenting input point clouds into horizontal and vertical planar surfaces using the PCL implementation of RANSAC. The 3D models were compact representations of the segment shapes in a BIM format like IFC (Industry Foundation Classes). Macher et al. [24] suggested a semi-automatic solution. After clustering the point cloud into floors using a histogram describing the distribution of points along the *Z*-axis, they performed region-growing segmentation on the 2D projection of each floor to identify room spaces. Based on the orientations and size criteria, planar surfaces previously segmented with Maximum Likelihood Estimation SAmple Consensus (MLESAC) were classified as walls, ceilings, and floors. The final model was generated as a volumetric representation of the architectural components (walls, ceilings, and floors) in IFC format. Using information from indoor scans, Murali et al. [12] generated 3D BIM models of one-floor buildings with hierarchical semantic annotations for individual rooms. They used voxel representations to filter input datasets, then a wall detection process was performed with RANSAC, followed by room layout detection and semantic model generation. Previtali et al. [25] formulated the reconstruction process as a labelling problem of structural cells in a 2D floor plan. They used RANSAC to extract potential indoor primitives. Then, a refinement step was performed to reduce under- and over-segmentation issues and get the final architectural component. A ray-tracing algorithm was utilized to identify openings and occluded regions in each segment. The final model was generated as a set of volumetric components with interconnections in IFC format.

Certain methods employ hierarchical labeling techniques to classify and label various components of buildings. These approaches typically involve a step-by-step process that begins with the detection of building levels, followed by the identification of rooms, walls, floors, and ceilings within each room cluster on the detected levels. Adan and Huber [26] developed a process to automatically generate BIM models of building rooms from a set of indoor 3D point clouds recorded at various locations in the room. Using information from the room scans, they automatically identified planar walls, floors, ceilings, and any significant rectangular openings. The method can be used to generate a complete and compact semantical-rich 3D model when applied to all the rooms in a building. Considering that the positions from which input scans were obtained are known and the environment is composed of multiple rooms bound by vertical walls, Mura et al. [27] extracted vertical planar blocks as candidates for wall structures in an occlusion-aware process. An automatic room detection process was performed by projecting candidate walls onto the XY plane from the raw constellation form. Then, accurate wall geometries were computed for each room. Finally, the model was created by intersecting wall planes with floor and ceiling planes. Assuming input point clouds consist of rooms, each scanned from one position, Ochmann et al. [28] used a diffusion process to perform room segmentation. Vertical structures detected from the segments were used to build a graph representing the ground constellation of the building, where the edges were considered walls. To enrich the model, they added an opening detection step for wall segments. After estimating the height of the building using a point distribution diagram, Jung et al. [8] projected a point cloud onto the XY plane. The room segmentation was conducted on 2D representations using information about spaces between rooms and door connections. The reconstruction process was performed on individual rooms by detecting permanent architectural components like walls, openings, and floors. Ochmann et al. [29] formulated the 3D model generation from point clouds as a linear integer optimization problem. After detecting planar surfaces, they applied Markov Clustering to cluster the pre-segmented blocks into separate rooms. With the help of the room-segmented point cloud, planes were refined and classified as candidates for vertical wall or horizontal slab surfaces. The interconnections between planes were determined by interpreting them as infinite planes. Then, planes were classified as belonging to rooms or the outside space by solving an optimization problem. The final model was obtained by exporting geometric and semantic information into IFC format.

Recently, learning and stochastic methods have been used to identify architectural components and their interconnections in point clouds. Khoshelham and Díaz-Vilariño [30] modelled 3D representations of indoor spaces by iteratively placing, connecting, and merging cuboid shapes detected from point clouds. Using a parametric shape grammar, they automatically learned interconnection rules between predetermined structures and generated 3D models of the interior spaces in the form of volumetric solids. Bassier et al. [31] used a voxelized representation of the input point clouds to extract planar patches. Then, clusters were classified as architectural components (walls, ceilings, floors) with a pre-trained set of Support Vector Machines (SVM). The reconstruction process was carried out from partial wall reconstructions, which consist of establishing relations between wall segments and representing them with BIM objects. After the wall reconstruction, rooms were reconstructed using information from the ceilings, floors, and partial wall objects. Finally, the topology of the walls was computed using information about the intersections between the partial walls and room repartition. Tran and Khoshelham [32] proposed a progressive stochastic approach. After segmenting the input point cloud into a set of volumetric cells as an arrangement of potential permanent architectural structures, the geometry of each shape was represented with a boundary representation, while the semantic information was used to indicate if the shape was navigable. At the model configuration stage, they defined transition steps that aided in optimizing the representation of the structures and global model. To find the most suitable model of the building described by the input point cloud, they adapted the reversible jump Markov Chain Monte Carlo with the Metropolis-Hastings algorithm. To improve the reconstruction result, Coudron et al. [33] performed a scene completion process using deep learning (an adaptation of the ScanComplete network). After the semantical completion of the input point cloud, objects labelled as clutter were removed, while the planar primitives representing the permanent structures were extracted. The 3D semantic model was generated with Blender. To find out how existing 3D BIM models could be used for improving conventional as-built modelling procedures, Ma et al. [34] introduced a point cloud-generating process from existing BIM models. The synthetic scans were then used to train a deep neural network designed for point cloud semantic segmentation. Such a well-trained model can be a solution for extracting geometric and semantic information while reconstructing 3D models of buildings from point clouds.

Some proposed solutions [2,18] generate a global 3D model without taking into account the presence of distinct architectural components, which leads to results far from reality. Other approaches [17,20,23] proceed by segmentation of the point cloud, followed by a classification step to assign each segment to a specific class (wall, floor, ceiling, facade, etc.). To enhance this critical phase of identifying the architectural components, we have developed specialized methods aimed at refining and ensuring a more faithful and accurate representation of the architectural components.

## 3. Methods

**Figure 1:**
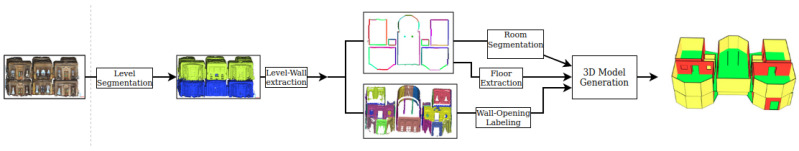
Proposed pipeline for the reconstruction of 3D models from indoor point clouds.

Automatically generating 3D semantic models from indoor point clouds is probably the fastest and cheapest approach for AEC professionals to obtain BIM models of existing buildings. The rendering of such processes depends not only on the quality of the point clouds but also on the ability of the process to identify architectural components and their interrelationships. In this section, we describe a pipeline used to generate 3D semantic models of buildings from indoor point clouds. We used specialized methods to detect different kinds of architectural elements (building floors, walls, ceilings, grounds, rooms, and openings) in the proposed pipeline. The flowchart in Figure 1 illustrates the main steps of the pipeline. The proposed pipeline takes as input the indoor point clouds of buildings. Without pre-processing, segmentation is performed to extract the potential floors of the building. For each floor block, the walls are then identified, followed by the extraction of the openings in each wall segment and the detection of different rooms and grounds. Once the information related to the different structural elements is extracted, the data is organized hierarchically and represented in a semantic 3D model in IFC format.

### 3.1. Level Segmentation

**Figure 2:**
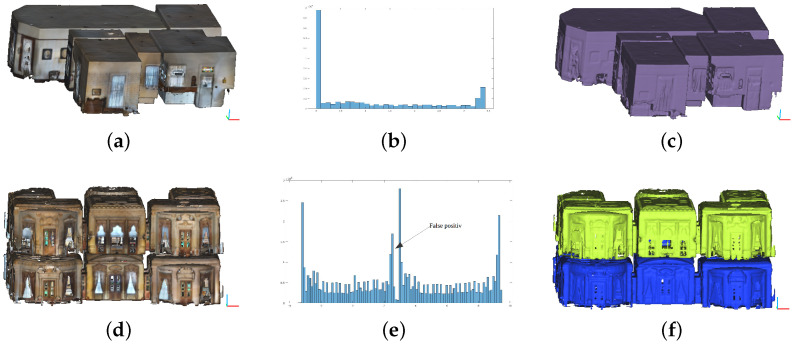
Results of level segmentation on two point clouds. (**a**,**d**) Raw point clouds. (**b**,**e**) Z-histograms of the raw point clouds. (**c**,**f**) Results of the segmentation process.

The Pipeline takes as input the indoor scan of a building. The point cloud is assumed to be aligned in XYZ space so that wall components are vertically parallel to the *Z* axis while structures such as floors and ceilings are horizontally aligned to the XY plane. For the segmentation of the input point cloud into potential levels of the building, we have opted for the use of a simple distribution histogram of the scan points on the *Z* axis. This approach is simple and gives satisfactory results [24,30]. Since our point clouds are scanned at the real scale we set the bin size to 0.1 m. Once the histogram is generated, the picks correspond to the ceilings or the grounds. From the first pick, all points located between two consecutive peaks are clustered as potential building floors. Since the distance between consecutive peaks naturally describes the height of the floors walls, close peaks (less than 10 bins) are considered false positives. Figure 2 shows two point clouds with corresponding Z-histograms and clustering results. The diagram in Figure 2e illustrates a false positive case. It can be clearly seen that the segmentation results identify the floors of the buildings faithfully.

### 3.2. Wall Detection

The wall extraction process is conducted using an extension of the 2D-grid approach proposed in [13] at each building level. In this study, a 2D ground projection is proposed to detect walls in unorganized indoor point clouds. The method takes the indoor point cloud of a single-story building as input. The point clouds are first segmented into horizontal slices along the walls. Then, from each segment, a binary N×M 2D grid is built. The matrix is large enough to contain the 2D projection of the initial point cloud onto the ground. Once the grids are extracted, they are locally merged into a matrix of the same dimension in order to obtain a reliable 2D constellation of the walls. Finally, a deterministic algorithm using continuous horizontal or vertical segments in the grid is employed to extract walls.

#### 3.2.1. Layers Segmentation

**Figure 3:**
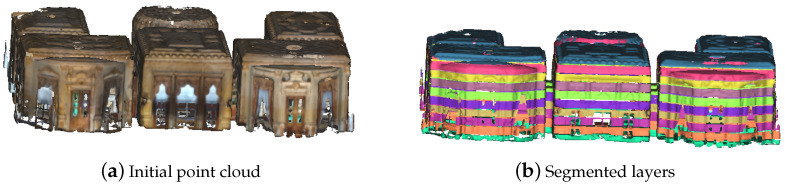
10-Layer segmentation of the second floor of the building in Figure 2d.

As stated in Section 3.1, the walls are assumed to be vertically aligned along the Z-axis in the global coordinate system XYZ. The layers are sliced in the XY plane between the lowest and highest points along the Z-axis of the bounding box. Let minZ and maxZ be the minimum and maximum coordinate values along the Z-axis of the initial point cloud. Let *n* be the number of layers, $L=\{L_{1},L_{2},\cdots ,L_{n}\}$ be the subset of the *n* layers and Cl be the initial point cloud. $L_{i}=\left\{P\left(x,y,z\right)\in Cl/minZ+\left(\left(maxZ-minZ\right)/n\right)\cdot \left(i-1\right)<=z<minz+\left(\left(maxZ-minZ\right)/n\right)\cdot i\right\}$. Figure 3 illustrates a division with n=10 applied on the second floor of the building in Figure 2d.

#### 3.2.2. 2D-Grid Generation

**Figure 4:**
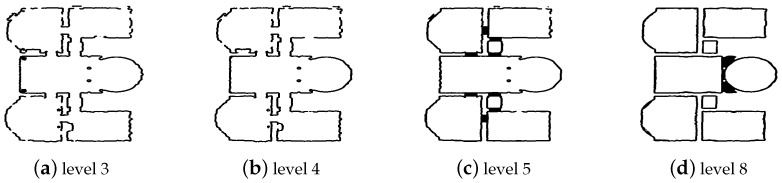
2D grid representation of layers $L_{3},L_{4},L_{5}$ and $L_{7}$ from the segmentation in Figure 3b.

The 2D grid representation of the initial point cloud is obtained by merging grid layers from the segmentation. Let $L_{i}$ be a layer and $MinZ_{i}$ the smallest value of *Z* coordinates indicated by the bounding box around $L_{i}$. $L_{i}$ is first projected onto the lower horizontal plane (all the *Z*-values of the $L_{i}$ points are turn into $MinZ_{i}$). After projection, an N×M grid matrix large enough to cover the entire base of the initial point cloud in the XY plane is generated. Let *d* be the density of the point cloud (the average distance between the points in Cl), P1(Xmin,Ymin,Zmin), P2(Xmax,Ymax,Zmax) are the lowest and highest points of the bounding box around Cl. *N* and *M* are defined as follows: N=(Xmax−Xmin)/d, M=(Ymax−Ymin)/d. Let $Gr_{i}$ be the grid generated from Layer $L_{i}$. The cell $Gr_{i}\left(k,j\right)=1$ if $NearestNeighbour(L_{i}^{\prime },\left(\left(k-1\right)\cdot d+minX,\left(j-1\right)\cdot d+minY,i\cdot minZ_{i}\right),d)>=threshold$. $L_{i}’$ is the projection of $L_{i}$ onto the lower horizontal plane, k∈{1,⋯,N} and j∈{1,⋯,M}. NearestNeighbour(Cl,P,d) returns the number of points of Cl close to points *P* in a radius *d*. Some 2D grids resulting from the segmentation in Figure 3 are illustrated in Figure 4.

**Figure 5:**
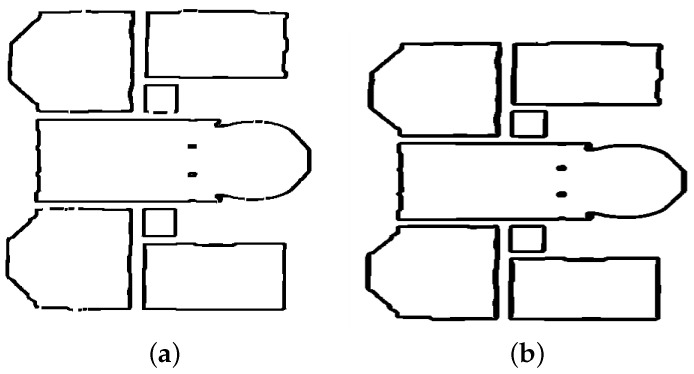
Final grids: (**a**) Grid obtained by merging $Gr_{2},Gr_{3},Gr_{4},Gr_{5},Gr_{6},Gr_{7}$ and $Gr_{8}$, (**b**) Grid after dilatation.

Once all the grids are computed, the merge is executed. Some conditions were defined to eliminate inconsistent grids. Based on the experiments, it was observed that the 2D representation of the wall constellation usually occupies less than 2/5 of the floor space of buildings and is generally greater than 1/10. Let $S=\{Gr_{1},Gr_{2},\cdots ,Gr_{n}\}$ be the set of grids generated from the *n* layers. $\forall Gr_{k}\in S,k\in \left\{1,\cdots ,n\right\},U=\sum_{i=1}^{N}\sum_{j=1}^{M}Gr\left(i,j\right)$. If U>=2/5·(M·N) or U<=1/10·(M·N), $Gr_{k}$ is eliminated. Let $R=\{Gr_{1},\cdots ,Gr_{l}\}$ be the set of remaining grids, Gr be a N×M grid. Gr(i,j)=1 if $\sum_{k=1}^{l}Gr_{k}\left(i,j\right)>=3/4l$. Figure 5a shows an example of a grid obtained by merging grids $Gr_{2},Gr_{3},Gr_{4},Gr_{5},Gr_{6},Gr_{7}$ and $Gr_{8}$.

#### 3.2.3. Wall Extraction

The wall extraction is executed using the concept of continuous segments in the row or column directions of the grids. Before performing the detection, a dilation step is conducted on the grid to fill gaps between the cells. The dilatation is done in the row and column directions based on the maximum distance between two non-zero cells of the same row or column. Let Gr be the binary grid of the initial point cloud, Max_d be the maximum filling distance between two non-zero linear cells, and $Gr(i,j_{1})$ and $Gr(i,j_{2})$ be two non-zero cells of row *i*, with $j_{1}<j_{2}$. If $j_{2}-j_{1}<=Max\_d$ and $\forall k\in ]j_{1},j_{2}[\;Gr\left(i,k\right)=0$, set all Gr(i,k)=1. The same process is conducted in the column direction. See Figure 5b.

**Figure 6:**
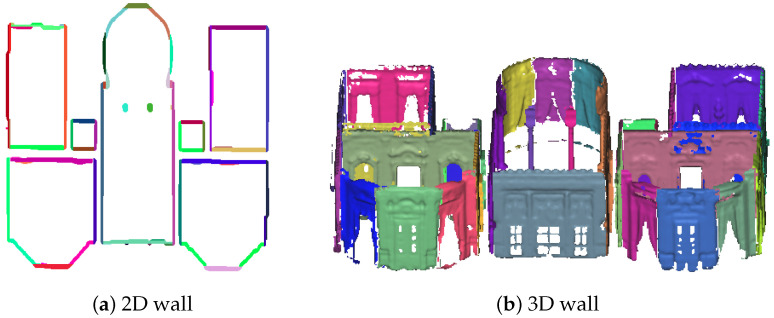
Wall detection result on the point cloud. (The image in Figure 5b has been rotated).

Let $A=Gr(i,j_{1})$ and $B=Gr(i,j_{2})$ be two non-zero cells of row *i* with $j_{1}<j_{2}$. [AB] is considered to be a continuous segment in the row direction if $Gr(i,j_{1}-1)=0$ and $Gr(i,j_{2}+1)=0$ and $\forall k\in ]j_{1},j_{2}[\;Gr\left(i,k\right)=1$. The cases where $j_{1}=1$ and $j_{2}=M$ don’t need testing because some null columns and rows were added to the grid. Once continuous segments have been identified, a cumulative algorithm is executed in the column or row direction to accumulate continuous segment blocks representing the walls in 2D. The 2D representations are then projected into 3D space to extract the 3D wall segments of the initial point cloud. Figure 6 illustrates the 2D and 3D results of the wall detection process on the second level of the building in Figure 2d.

**Figure 7:**
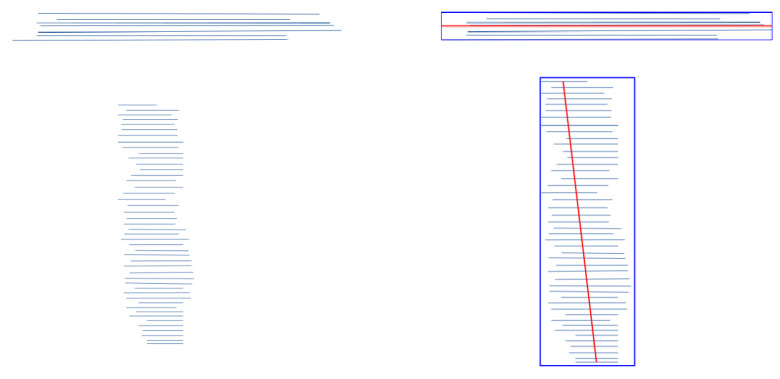
Identification of the representative segments. The red lines in the images of the second column are representative segments.

In order to improve the representation of wall blocks in the final model, we introduced the concept of representative segments. They are single segments representing wall blocks in two dimensions. These representative segments are designed from the blocks of segments that constitute the walls in the global 2D-grid. Let $W=\{S_{1},S_{2},\cdots ,S_{m}\}$ be the set of segments constituting a wall *W*. $S_{i}$ can be written in the form Si=[AB], $A=Gr(i,j_{1})$ and B=Gr(i,j2), with $j_{1}<j_{2}$. $S_{i}$ can be represented by $j_{1}$ and $j_{2}$, its lower and upper bounds. We denote the set of lower indices of the $S_{i}$ segments by $Inf=\{L_{1},L_{2},\cdots ,L_{m}\}$ and the set of its upper indices by $Sup=\{U_{1},U_{2},\cdots ,U_{m}\}$. Let $J_{inf}=min\left(Inf\right)$ and $J_{sup}=max\left(Sup\right)$. The amplitude of *W* in the direction of segment orientation is $\left||W|\right|=J_{sup}-J_{inf}$. For the identification of representative segments, two cases are identified. The case where the number of segments constituting the block is lower than the cover distance of the block in the direction of segment orientation: ||W||>=m and the opposite case (||W||<m). The two cases are shown in Figure 7.

In the first case (||W||>=m), the representative segment is collinear to the longest segment of the block, covering the whole block length. Let $S_{k}=\left[A,B\right]$ be the longest segment of *W*, with $A=Gr(i,j_{k1})$ and $B=Gr(i,j_{k2})$. Let $S_{k}=\left[I_{1},I_{2}\right]$ be the representative of *W*, $I_{1}=Gr\left(i,J_{inf}\right)$ and $I_{2}=Gr\left(i,J_{sup}\right)$. Such a case is illustrated by the red segment in the image at the top right (see Figure 7). In the second case, the representative segment is the one that extends from the middle of the first block segment to the middle of the last block segment. See the red line in the image at the bottom right of Figure 7.

**Figure 8:**
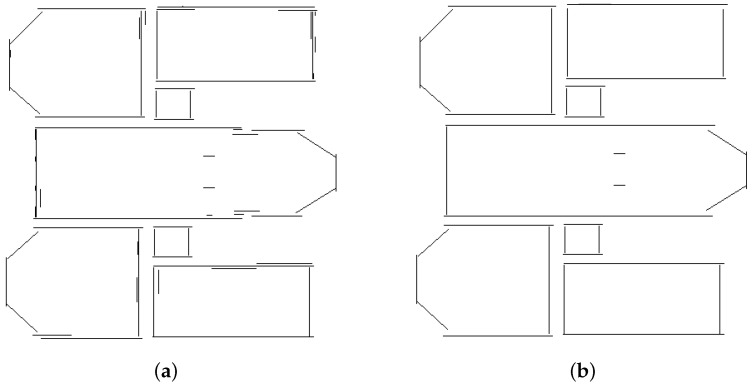
Improvement of the wall representation. (**a**) Representative segments, (**b**) Merging of the parallel segments.

Figure 8a shows the representative segments obtained from the blocks in Figure 6a. As can be seen, most of the segments representing connected walls appear to be disconnected from one another. A first operation consisting of merging parallel segments is executed to eliminate the insignificant segments. The result of the operation is illustrated in Figure 8b. After merging the parallel segments, the connections between neighboring walls are not correctly established, hence the need to update the representative segments.

The process of extending representative segments is designed to create definitive connections between neighboring segments. For each vertex of a given segment, two operations are executed.

1. Searching for the segment closest to the current vertex.
2. Segments updating

Let [AB] be one of the representative segments. The processing of vertex A is executed as follows: The first operation is guided by a maximum distance parameter (noted as *d*), which delimits the maximum distance to be taken into account. [AB] is first extended infinitely from the vertex *A*. Intersections of the half-line (AB] with the lines supporting the other segments are listed. Let $I=\{I_{\left[A_{1}B_{1}\right]},I_{\left[A_{2}B_{2}\right]},\ldots ,I_{\left[A_{n}B_{n}\right]}\}$ be the set of intersections. $I_{\left[A_{i}B_{i}\right]},i\in \left\{1,2,\ldots ,n\right\}$ represents the intersection of (AB] and the segment $\left[A_{i}B_{i}\right]$. $\left[A_{j}B_{j}\right]$ is considered as the closest segment to *A* if $||AI_{\left[A_{j}B_{j}\right]}\left|\right|<d$ and $||AI_{\left[A_{j}B_{j}\right]}\left|\right|=\arg\min_{i\in \{1,2,\ldots ,n\}}(||AI_{\left[A_{i}B_{i}\right]}\left|\right|$) and ($\left|\right|A_{j}I_{\left[A_{j}B_{j}\right]}\left|\right|<d$ or $\left|\right|B_{j}I_{\left[A_{j}B_{j}\right]}\left|\right|<d$ or $I_{\left[A_{j}B_{j}\right]}\in \left[A_{j}B_{j}\right]$).

**Figure 9:**
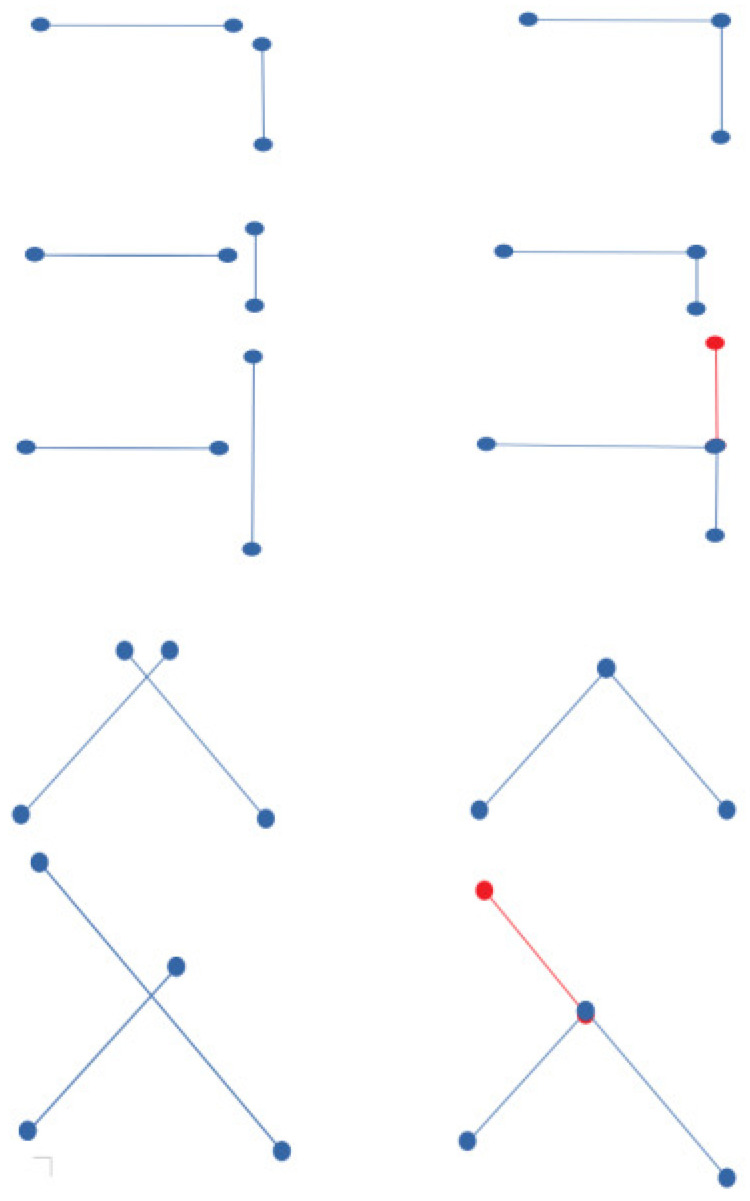
Illustration of representative segment configurations requiring updates. (**left**) are the initial configurations, and (**right**) are the states after updates. In certain cases, new segments can be created (red segments).

At the end of the first operation, if a segment $\left[A_{j}B_{j}\right]$ is selected, a second operation consisting of updating [AB] and $\left[A_{j}B_{j}\right]$ is executed. As depicted in Figure 9, several distinct cases can be discerned and can be categorized as $I_{\left[A_{j}B_{j}\right]}\in \left[A_{j}B_{j}\right]$ and $I_{\left[A_{j}B_{j}\right]}\notin \left[A_{j}B_{j}\right]$.

- $I_{\left[A_{j}B_{j}\right]}\notin \left[A_{j}B_{j}\right]$: $A=I_{\left[A_{j}B_{j}\right]}$, if $\left|\right|A_{j}I_{\left[A_{j}B_{j}\right]}\left||<|\right|B_{j}I_{\left[A_{j}B_{j}\right]}\left|\right|$, $A_{j}=I_{\left[A_{j}B_{j}\right]}$ else $B_{j}=I_{\left[A_{j}B_{j}\right]}$.
- $I_{\left[A_{j}B_{j}\right]}\in \left[A_{j}B_{j}\right]$: $A=I_{\left[A_{j}B_{j}\right]}$, if $\left|\right|A_{j}I_{\left[A_{j}B_{j}\right]}\left||<|\right|B_{j}I_{\left[A_{j}B_{j}\right]}\left|\right|$ and $\left|\right|A_{j}I_{\left[A_{j}B_{j}\right]}\left|\right|<d$, $Aj=I_{\left[A_{j}B_{j}\right]}$. if $\left|\right|A_{j}I_{\left[A_{j}B_{j}\right]}\left||<|\right|B_{j}I_{\left[A_{j}B_{j}\right]}\left|\right|$ and $||AjI_{\left[A_{j}B_{j}\right]}\left|\right|>d$, create a new segment $\left[A_{j}I_{\left[A_{j}B_{j}\right]}\right]$ and update $\left[A_{j}B_{j}\right]$ ($A_{j}=I_{\left[A_{j}B_{j}\right]}$). Repeat the same actions if $\left|\right|A_{j}I_{\left[A_{j}B_{j}\right]}\left||>|\right|B_{j}II_{\left[A_{j}B_{j}\right]}\left|\right|$.

**Figure 10:**
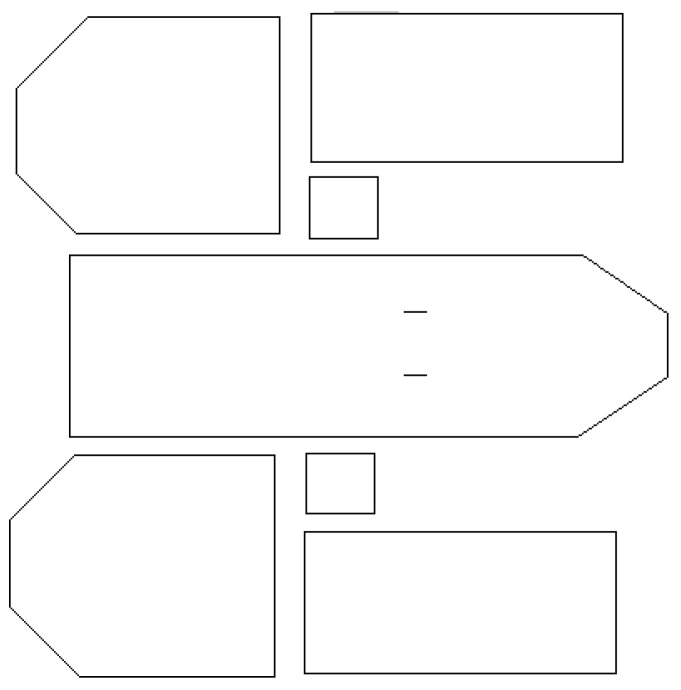
Final representation of the representative segments after the extension.

Figure 10 illustrates the final representation of the representative segments after the extension operation. As can be seen, the interconnections are clearly established.

### 3.3. Room Detection

In the 3D semantic model of a building, the identification of the different rooms constituting the building is capital. Such information is useful for the hierarchical organization of the structural components. It can also be used in indoor orientation and navigation systems. In this subsection, we propose a new method for room extraction in the indoor point cloud of a one-level buildings. The method uses a 2D map designed from the ground constellation of walls previously computed.

**Figure 11:**
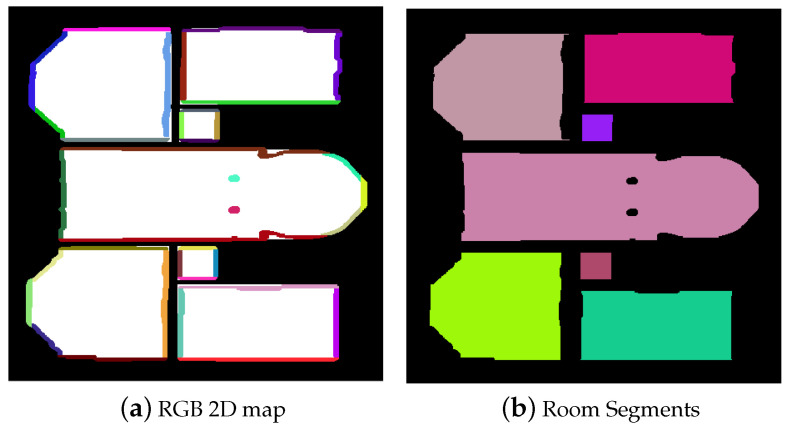
Room segmentation result.

The generation of the map is done using an iterative algorithm executed on the rows and columns of the RGB matrix representing the wall constellations (Figure 8d). It should be noted that the RGB values used to colour the walls have been randomly generated between 1 and 254. Any pixel of the matrix with a value of (255, 255, 255) is void. The map generation algorithm proceeds as follows: for each row and column of the images of the wall constellations, all consecutive sequences of pixels marked as empty from the beginning and end of the rows take the value (0, 0, 0). Figure 11a shows an example of such a map.

Once the map is generated, the room detection process can be conducted with a region-growing algorithm as in [35] or with an approach based on continuous segment accumulation as previously presented in the wall detection phase. In this work, we have used the second approach. Figure 11b shows a random coloring of the different rooms obtained from the room detection process. The same result can be obtained from a constellation with representative segments. A space is classified as a room if it is bounded by a set of interconnected segments, as described in Figure 8d.

### 3.4. Detection of Openings

To reconstruct the BIM model of the buildings with high precision and accuracy, it is imperative to efficiently extract all building components including openings(doors and windows) [36]. Since the openings are parts of walls, after extracting wall segments, the detection process is easier. We developed a two-step method for identifying openings in point clouds of building walls based on the 2D grid and continuous segment concepts previously employed for wall detection. Holes are first extracted from the wall segments. Then, a classification method is executed to separate holes representing windows and doors.

#### 3.4.1. Hole Extraction

**Figure 12:**
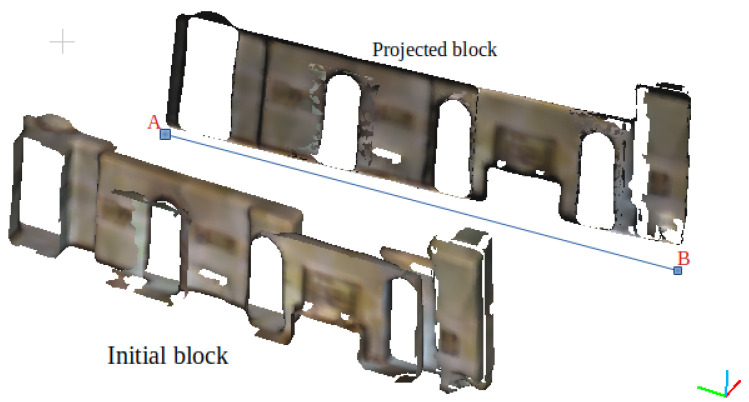
Wall block projection in the 2D plane passing through the representative segment [AB] and parallel to the *Z* axis. The projected block has been displaced.

The proposed method detects doors and windows that were open during the scanning process or those in which the door or window covers were ignored in the wall extraction operation. Thus, empty areas in the wall blocks were considered potential windows or doors. The extraction of candidate entities was done using a grid similar to that used for wall detection. The generation of the grid is executed as follows: Points constituting the wall are first projected in the 2D plane, passing through the representative segment and parallel to the *Z* axis. Figure 12 shows an example of such a transformation applied to a wall segment. The projection has been displaced to distinguish it from the original wall. A grid of size M×N large enough to cover the projected block (Cl) is generated on the projection plane.

Let $A(x_{1},y_{1})$ and $B(x_{2},y_{2})$ be the coordinates of the extremities of the representative segment in the XY plane (original point cloud environment). Let $Z_{min}$ and $Z_{max}$ be the minimum and maximum values of the *Z* coordinate in the set of points of the wall block. Let *d* be the density of the projected cloud point (the average distance between points). The MxN grid Gr (with $M=|Z_{max}-Z_{min}|/d$ and N=distance(A,B)/d) is generated in 3D space bounded by the points $S_{1}\left(x_{1},y_{1},Z_{min}\right)$, $S_{2}\left(x_{2},y_{2},Z_{min}\right)$, $S_{3}\left(x_{2},y_{2},Z_{max}\right)$ and $S_{4}\left(x_{1},y_{1},Z_{max}\right)$.

Let Gr(i,j) be the grid cell at the $i_{th}$ row and the $j_{th}$ column (i∈1,2,⋯,M and j∈1,2,⋯,N). The 3D coordinates (X,Y,Z) of the cell Gr(i,j) are computed as follow:

$$X=x_{1}+\left(j-1\right)/\left(N-1\right)\left(x_{2}-x_{1}\right)$$

$$Y=y_{1}+\left(j-1\right)/\left(N-1\right)\left(y_{2}-y_{1}\right)$$

$$Z=Z_{min}+\left(i-1\right)*d$$

**Figure 13:**
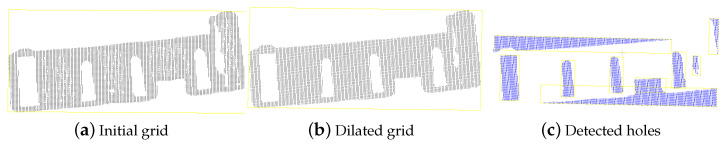
Hole extraction result.

Gr(i,j) is marked as occupied (Gr(i,j)=1) if NearestNeighbour(Cl,(X,Y,Z),d)>=1 otherwise Gr(i,j) is marked as empty (Gr(i,j)=0). Figure 13a presents a sample grid.

Once the grid is constructed, a dilation operation is performed to fill the excess empty cells created during grid construction before proceeding with the hole extraction process. The propagation principle is similar to that used for wall detection. Figure 13b presents a dilated version of the grid in Figure 13a. Hole extraction is performed using the concept of continuous segments. Instead of accumulating continuous segments of non-empty cells, we accumulate continuous segments of empty cells. We ignored segments extending along the entire grid length to reduce the impact of the empty blocks at the borders created during the 2D grid construction. Figure 13c shows holes in bounding boxes (yellow) detected from the dilated grid. All the detected holes are not necessarily doors or windows, as observed. Some result from partially incomplete reconstruction or blocks of empty cells generated by the 2D grid construction. To identify the openings (doors and windows) from the random holes, we opted to use a classification model.

#### 3.4.2. Deep Learning-Based Classification

Let $D=\{\left(X_{1},Y_{1}\right),\cdots ,\left(X_{N},Y_{N}\right)\}$ be our dataset with *N* observations, where each observation consists of a feature vector $X\in R^{p}$ and a target variable *Y*. Our dataset consisted of 1000 holes. Each hole feature, denoted as $X=(x_{1},\cdots ,x_{7})$, is represented as a disentangled vector with a dimension of p=7, capturing its distinct semantic characteristics. Its corresponding target label, Y, representing whether it is a door, window, or non-opening, is encoded using a one-hot encoding vector: (1, 0, 0) for doors, (0, 1, 0) for windows, and (0, 0, 1) for non-openings. The semantical characteristics constituting the feature vector are defined as:

- The size of the matrix. The number of rows and columns of the grid generated by the projection of the wall on the representative 2D plane. $x_{1}$ and $x_{2}$.
- The position of the bounding box around the hole in the grid. Four inputs are necessary: the numbers of the first and last line and the numbers of the first and last column (the size of the bounding box is computed from this information). $x_{3},x_{4},x_{5}$ and $x_{6}$
- The occupancy rate of the box. The percentage of cells marked as occupied in the bounding box around the hole. $x_{7}$.

In the dataset, 59.3% of the holes are classified as noise, 28.5% as doors, and 12.2% as windows. The holes were obtained from the wall extraction process applied to ten indoor point clouds. The data set was manually classified. Data augmentation was performed by manually segmenting some blocks to create windows and doors and hence improve the quality of the data set.

The objective of the classification is to learn a function or classifier $\psi_{\theta }:X\to Y$ capable of predicting the class of a hole according to its 7-features, i.e., learning the θ parameters. Using the dataset, parameters θ can be approximated by solving the following optimization problem:

(1) $$\hat{\theta }=\underset{\theta }{arg max}\sum_{i=1}^{N}\log P\left(Y_{i}|X_{i};\theta \right)$$

where the probability term *P* is defined as a softmax function and θ the learnable parameters. To model these parameters, we designed a deep neural network architecture with an input dimension of p=7 and an output dimension of n=3. The architecture includes two densely connected hidden layers with an intermediary dropout regularization layer. The activation function used in the hidden layers is linear, while the output layer employs softmax activation. During training, we employ the widely-used *Adam* optimizer to learn the weights of the network (i.e., the parameters θ) with the Cross-Entropy (CE) loss:

(2) $$L_{CE}=-\frac{1}{N}\sum_{i=1}^{N}\sum_{c=1}^{3}Y_{i}\left[c\right]\cdot \log\left({\hat{Y}}_{i}\left[c\right]\right)$$

where $Y_{i},{\hat{Y}}_{i}\in {\left[0,1\right]}^{3}$ are one-hot of ground truth and model’s predicted probability vector of the $i^{th}$ observation, respectively.

**Figure 14:**
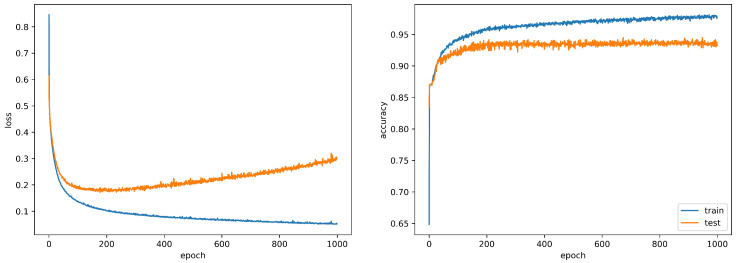
Loss and accuracy for train and test sets after 1000 epochs. A 5-fold cross-validation has been performed.

Figure 14 presents the loss and accuracy performance during training and testing. An early stopping regularization was used to prevent the model from overfitting. Due to the imbalanced data distribution, additional evaluation of the model’s performance was conducted using the f1-score metric. The results of this evaluation indicate that the significant imbalanced distribution in the dataset has introduced a slight bias towards classes that are more prevalent in the data. This can be observed by examining the classification report presented in Table 1. Despite such an imbalanced distribution, the model accurately predicts classes with low representation.

### 3.5. Model Generation

The last step in the pipeline consists of translating the geometric information of the architectural components previously detected into a BIM model. The BIM model is generated in IFC format. IFC is an open, vendor-neutral data exchange format developed by the international organization buildingSMART to facilitate collaboration between different fields involved in AEC projects (information sharing) [37]. In IFC models, the building is decomposed into architectural components and spaces described in an object-oriented approach using semantic and geometric information.

**Figure 15:**
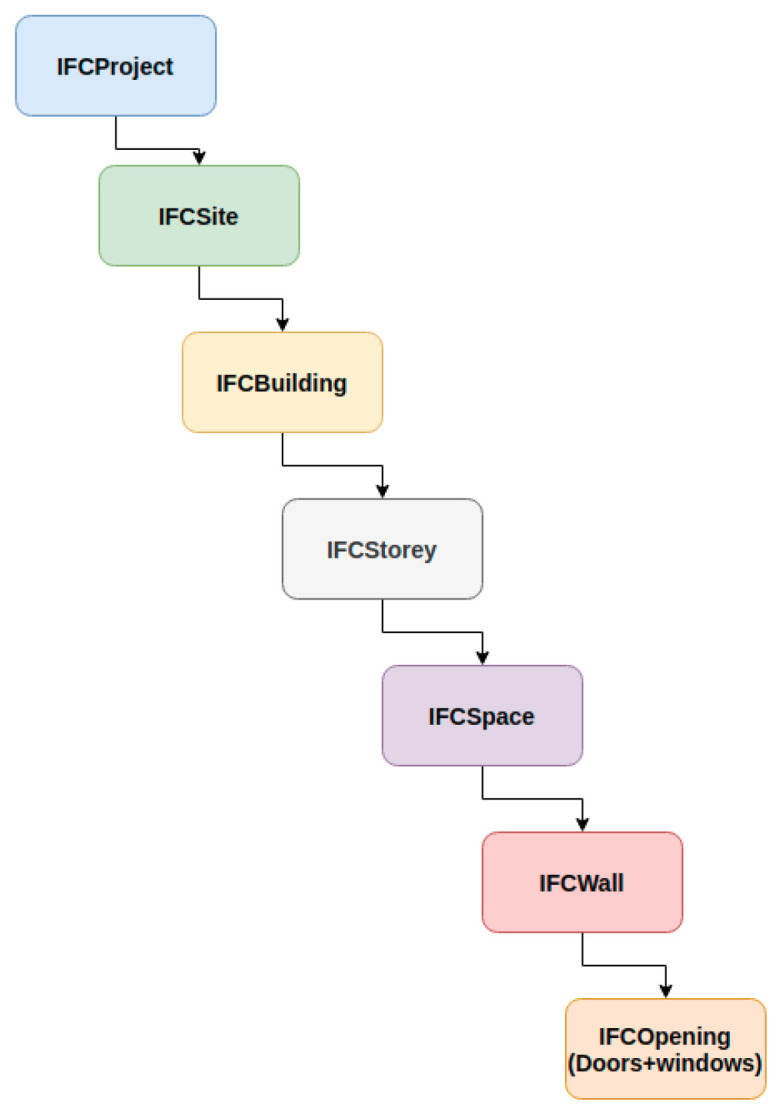
Global hierarchical organization and relationships between structural elements in a simple IFC file.

Each architectural element previously detected is represented with the corresponding class in IFC format. Then, based on the relationships between the components, the hierarchical structure representing the complete building is generated. Figure 15 shows a hierarchical representation of a building in the final IFC model. The essential information for representing the architectural elements is the coordinates of points describing the object in a 2D plane, the position in 3D space, the height, and the parent object in the hierarchy. Based on the hierarchy defined in Figure 15, the components are generated as follows:

The object IFCSite represents the spatial environment (area) on which the building is designed (It is possible to have several buildings on the same site). The most important information at this stage is the 2D coordinates of the points delimiting the space in the XY plane. Let $X_{min}$, $X_{max}$, $Y_{min}$, and $Y_{max}$ be the minimum and maximum values of the coordinates of the points constituting the initial point cloud on the *X* and *Y* axes. The Site is the space delimited by the points $S_{1}\left(X_{min},Y_{min}\right)$, $S_{2}\left(X_{min},Y_{max}\right)$, $S_{3}\left(X_{max},Y_{max}\right)$, and $S_{4}\left(X_{max},Y_{min}\right)$. IFCSite has IFCProject as the parent object.

Building (IFCBuilding). The essential information is the coordinates of the points delimiting the space of the site where the building is located. The building space is delimited by the points $S_{1}$, $S_{2}$, $S_{3}$, and $S_{4}$ previously defined. The building has the site as the parent object.

**Figure 16:**
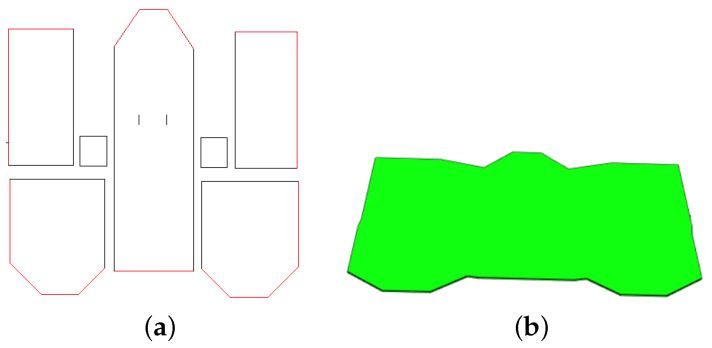
Ground extraction of the 2 story building. (**a**) 2D Ground contour in red, (**b**) 3D representation of the ground in the final model.

Floor (IFCBuildingStorey). IFCBuildingStorey defines the building floors. The essential information at this level is the 2D coordinates of the points delimiting the space occupied by the floor in the XY plane. The process of identifying these points is conducted using representative segment constellations. A map similar to the one in Figure 11a is generated. This map makes it possible to identify the outer cells belonging to the representative segments. The red lines in Figure 16a indicate the sequence of the ground points in 2D generated from the constellation in Figure 8d. After the points are identified, the nearest neighbor algorithm is used to extract the required sequence of points. In addition to the list of points, the 3D position of the object IfcStorey is defined. Let h(i) be a function used to define the height of the floor *i*. The position of the first floor is given by the coordinate point $P_{1}\left(0,0,0\right)$, and the position of level i!=1 is given by the $P_{I}\left(0,0,\sum_{i=1}^{I-1}\left(h\left(i\right)\right)\right)$.

The same information is used to represent the ground and ceiling of the current building floor. The 2D shape is described using the sequence of points previously extracted in Figure 16a. The ground thickness is defined as 0.1 m. The difference between the ground and ceiling of a building floor is their positions along the Z-axis in 3D space. For floor *i*, the position of the ground is defined by $P_{i}\left(0,0,h\left(i-1\right)\right)$ and the ceiling is $Pl_{i}\left(0,0,h\left(i\right)\right)$ (with h(0)=0). Figure 16b shows an example of the ground representation in the model.

**Figure 17:**
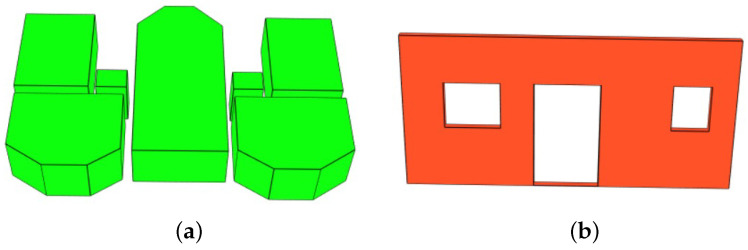
Space and wall representations. (**a**) Top floor of the 2-story building. (**b**) Wall with a door and 2 windows.

Space (IFCSpace). IFCSpace represents an empty volume (room). The points describing the volume projection in the XY plane are the intersections between the wall representative segments delimiting the rooms. The position and height of a room in 3D space are the same as the corresponding level. Figure 17a shows examples of rooms in the model.

Wall and opening. The walls are described with the object IFCWall. The coordinates of the points describing the projection of a wall in the XY plane are derived from the representative segment. Let *d* be the general thickness of the walls, $A(X_{A},Y_{A})$ and $B(X_{B},Y_{B})$ the coordinates of the representative segment in the XY plane. Let Y=aX+b be the equation of the line (AB). Y=aX+b+d/2 (denoted $\left(D_{1}\right)$)and Y=aX+b−d/2 (denoted $\left(D_{2}\right)$) are the equations of the parallel lines to (AB) located at distance d/2 on either side of (AB). $Y=-X/a+Y_{A}+X_{A}/a$ (denoted $\left(D_{3}\right)$) and $Y=-X/a+Y_{B}+X_{B}/a$ (denoted $\left(D_{4}\right)$) are the lines perpendicular to $\left(D_{1}\right)$ and $\left(D_{2}\right)$ and passing through *A* and *B*, respectively. The points describing the shape of the wall on the XY plane are the 4-intersection points between $\left(D_{1}\right),\left(D_{2}\right),\left(D_{3}\right)\mathrm{and}\left(D_{4}\right)$. The position of a wall located at level *i* and its height correspond to $P_{i}$ and h(i), respectively. The openings belong to the walls. Represented with the object IFCOpening, they are either doors or windows. In order to align openings, the window heights $H_{w}$, are aligned at the same height as the door heights $H_{d}$. Let P(0,0,Z) be the position of a wall. The windows on the wall are located at the position $P_{w}\left(0,0,Z+H_{w}\right)$ and doors at $P_{d}\left(0,0,Z+H_{d}\right)$. The heights of the openings are standardized. Figure 17b shows a wall with openings (a door and two windows).

### 3.6. Computational Costs and Scalability

The pipeline is a combination of independent methods and procedures that can be implemented and executed hierarchically and sequentially. Suppose the input point cloud has size *n* then:

1. Segmentation into potential levels of the building: This step runs in the worst case in 2×O(n) time, since it mainly involves the construction of a histogram and its traversal. In terms of memory space, only one copy of the point cloud is needed.
2. Wall detection: As mentioned in [13], this step runs in O(nlogn) time and requires, in the worst case, a memory capacity at least twice the size of the initial point cloud. For horizontal layer segmentation, one copy is sufficient. If the matrix size is $K_{1}\times K_{2}$ and we have $K_{3}$ layers, the memory capacity required to obtain the final wall constellation matrix will be $\left(K_{3}+1\right)\times \left(K_{1}\times K_{2}\right)$. Since $K_{1},K_{2},K_{3}\ll n$, a memory capacity twice the size of the initial point cloud is sufficient.
3. Extraction of openings:This step can be reduced to simply extracting holes, as classification is done in constant time O(1). For a given wall segment, the hole extraction is done in the worst case in time O(W), where *W* represents the segment size. If the building has $N_{1}$ levels and each level has $N_{2}$ walls, the total execution will be done in time $N_{1}\times N_{2}\times O\left(W\right)$. In the worst case, W∼n and $N_{1}\times N_{2}\ll n$, which gives an approximate execution time of O(n). For processing each block, a memory space equal to the size of the initial point cloud is sufficient. Since the execution is sequential, this space can be reused.
4. Extraction of floor contours and rooms: This step depends mainly on the size of the matrix. The time and memory space required are negligible compared to the initial point cloud, which generally contains several million points.
  In summary, the approximate execution time of the pipeline is the sum of the times of the different steps: 2×O(n)+O(nlogn)+O(n)∼O(nlogn). Regarding the memory space, since the operations are executed sequentially, the space can be reused. Thus, a memory capacity equivalent to twice the size of the initial point cloud is sufficient.

## 4. Implementation and Results

### 4.1. Implementation

We utilized the data structures offered by CCLib [38] to execute point cloud manipulations. Matrix operations were conducted using OpenCV [39], while the generation of the IFC was achieved using the IfcOpenShell library [40]. In addition to being open source, IFCOpenShell provides all the necessary C++ data structures to represent the previously described objects in the IFC format, specifically version IFC 2 × 3. Furthermore, the object-oriented nature of the C++ language simplifies the hierarchical representation of the final model.

The implementation of the *IFCProject* node is achieved through the *createIfcProject()* function, which requires at least two parameters: the project name and an object of the *ifc2x3::ExpressDataSet* class. The object is progressively constructed throughout the model generation process and contains the geometric and semantic information of the model elements. The *ifc2x3::ExpressDataSet* object is sequentially passed as a parameter to all the functions involved in the model construction. Optionally, *createIfcProject()* can also include information about the file owner and the units of measurement used in the model. The creation of a site is accomplished using *createIfcSite()*, which requires parameters such as the sequence of points that define the site’s 2D space, the site name, and the project to which the site belongs. *IFCBuilding* is implemented with *createIfcBuilding()*, which considers as parameters the building name, the associated site, and a list of 2D coordinates that define the building’s constructed space on the site. The creation of floors in buildings (*IFCStorey*) is carried out using *createIfcStorey()*. In addition to the list of 2D points that define the floor’s projected space on the ground and the identity of the building to which the floor belongs, a crucial piece of information required by *createIfcStorey()* is the position of the floor in 3D space. The creation of the floor and ceiling slabs is accomplished using *createIfcSlab()*. Rooms are created with *createIfcSpace()*. Walls are generated using *createIfcWall()*. An important parameter for *createIfcWall()* is a boolean value that specifies if the wall has openings. Doors and windows are created using *createIfcWindows()* and *createIfcDoor()*, respectively. Once all the elements are constructed, the *ifc2x3::ExpressDataSet* object is passed as a parameter to *ifcWriter()* for the generation of the corresponding IFC content.

**Figure 18:**
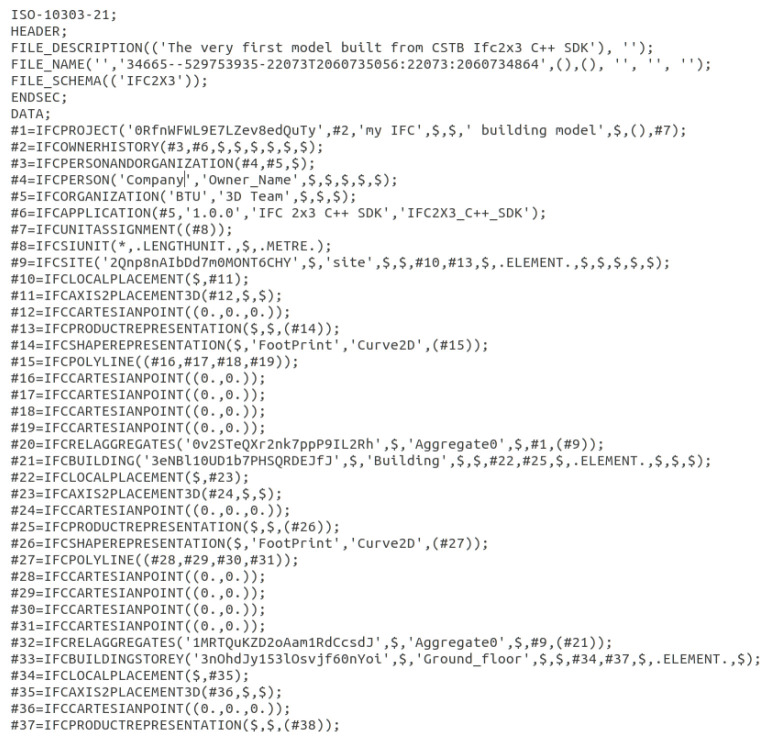
Illustration of IFC file content header.

IFC files provide a common structure for representing and storing building elements and infrastructure data in a way that they are readable and interpretable, thereby facilitating interoperability and information exchange between different software applications used by stakeholders in an AEC project. IFC is based on the EXPRESS data modeling language, which defines the schema and rules for representing building information. In IFC, building elements are considered entities described using attributes and relationships. Each entity, attribute, or relationship has a reference (#number) used to identify and construct buildings’ hierarchical structures. Figure 18 shows the header of an example IFC file. It is a simple block of sequential references that is easy to interpret.

**Figure 19:**
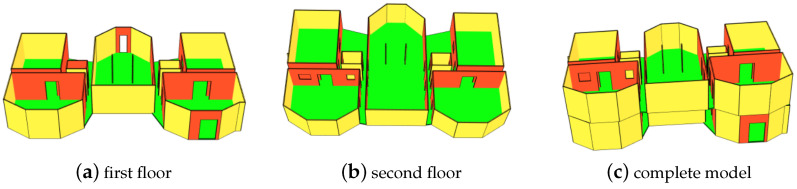
Final 3D model of the multi-levels building in Figure 2d.

Once the model is generated, it can be visualized using an IFC viewer. An IFC viewer provides a graphical interface for navigating through the BIM stored in IFC files. It allows users to visualize 3D models, explore the building’s components, and access associated metadata. In addition to allowing 2D or 3D visualization, an IFC viewer also offers features such as object selection, clash detection, measurements, annotation, and data extraction. Most AEC applications available on the market (Autodesk, Solibri, BIMcollab, etc.) include a BIM viewer. In this work, we used the one proposed in IFCengine. Figure 19 illustrates the complete visualization of the BIM model of the building in Figure 2d. To improve rendering, openings have been manually created on the segments after the wall detection step.

### 4.2. Results

We tested the pipeline’s efficiency and robustness on indoor point clouds of buildings from the Matterport3D datasets [41]. The Matterport3D acquisition equipment is a rotating system with three color cameras and three depth cameras fixed on a tripod. The reconstruction of point clouds of buildings is executed as follows. Six panoramic images are recorded in different directions. The resulting 18 panoramic images are stitched together to obtain a suitable panoramic view. Information on the depths from the depth cameras was integrated to synthesize depth maps, which were then aligned to the panoramic images to generate point clouds. Each scene was taken within a radius of 2.5 m. The point clouds of the scenes were finally merged using registration processes to form complete building point clouds. The Matterport3D scans consist of millions of points covering an average area of 1500 m^2^.

**Figure 20:**
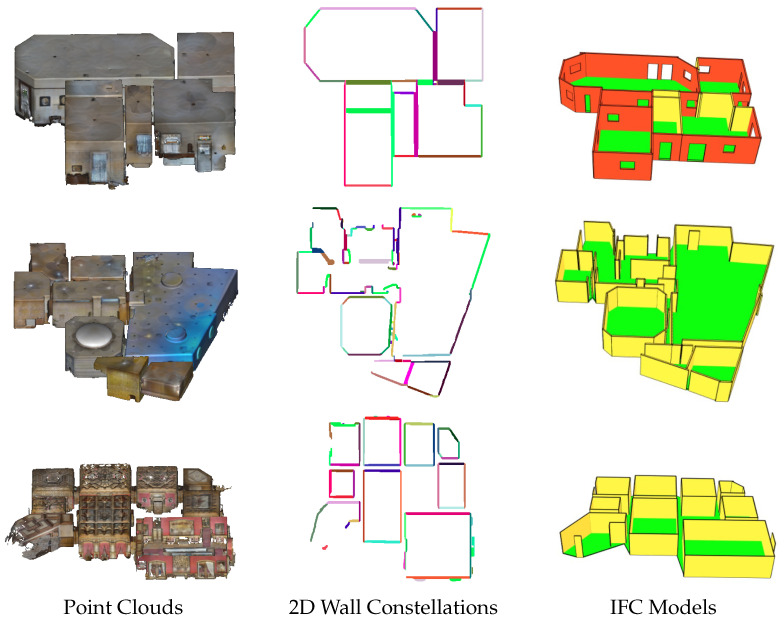
Results of the three point clouds. The second column presents 2D wall constellations. The third column presents the results of the IFC model.

Figure 20 presents the results of the reconstruction obtained on 3-point clouds (the second column presents wall constellations on the ground). As mentioned earlier, most Matterport3D point clouds were scanned with doors and windows closed, hence the absence of openings in some models. The results in Figure 4 demonstrated the effectiveness and reliability of the proposed pipeline. The pipeline successfully extracted architectural elements, identified structural components, and generated accurate 3D semantic models of buildings from indoor point clouds. The architectural element extraction process achieved high precision and recall values, indicating the pipeline’s ability to accurately identify walls, floors, ceilings, openings, and rooms. The structural component identification stage exhibited high accuracy and completeness, ensuring the consistency of the generated models.

We conducted a thorough visual inspection to assess the quality and fidelity of the generated 3D semantic models. The inspection revealed our models closely resembled the ground truth models, indicating high fidelity and accuracy of the pipeline. The models effectively captured both the geometric properties, such as the shape, dimensions, and spatial relationships of the architectural elements, as well as the semantic properties, including their intended functionalities and roles within the building. This comprehensive representation of both geometric and semantic properties allows for a wide range of applications in various domains, including architecture, engineering, construction, gaming, augmented reality, and indoor navigation. The generated models serve as reliable digital replicas of the real-world buildings, facilitating improved understanding, communication, and decision-making. Through accurate architectural element extraction, precise structural component identification, and the generation of faithful 3D semantic models, our pipeline provides a powerful solution for automatic 3D model generation from point clouds. However, upon close examination of the results, it is apparent that the reconstruction exhibits deficiencies in some cases. The approach employed to represent architectural elements fails to capture non-rectilinear shapes. Such a case is illustrated in the model depicted in Figure 19, where circular walls, although detected during the wall extraction process (see Figure 6b), are erroneously represented as rectilinear in the final model.

## 5. Conclusions

In this paper, we presented a comprehensive pipeline for the automatic generation of 3D semantic models of buildings from indoor point clouds. The proposed pipeline successfully extracted architectural elements and generated accurate and consistent 3D semantic models. The results obtained from our pipeline demonstrated the effectiveness and reliability of our approach. The pipeline addressed the limitations of manual reconstruction processes by automating the generation of 3D models from building scans. The building point cloud levels are segmented using a histogram. The walls are extracted using a constellation derived from merging representative 2D grids. The wall constellation allows the extraction of the rooms and contours of the ground. A deep learning-based classifier is used to recognize windows and doors from wall segments. By leveraging specialized detection methods for each architectural class and employing a hierarchical approach, we ensured optimal classification and reliable reconstruction. The proposed pipeline consistently generated reliable results, demonstrating high levels of accuracy, efficiency, and reliability.

Despite the quality and precision of the results of the different extraction methods, the representation of certain structural elements in the final models did not reflect the ground truth. The reconstruction of complex structures such as circular walls was not considered in the generated models. However, the approaches and methods implemented in this work can be used in other pipelines or improved to obtain more efficient results. In conclusion, the proposed pipeline presents a novel option to automate the detection of structural components in indoor point clouds.

## Figures and Tables

**Table 1:** Model performance. The classification report indicates the performance metrics for the different classes are reasonable, despite the imbalanced distribution of data among the classes.

	Precision	Recall	f1-Score	Support
**door**	0.95	0.86	0.90	80
**window**	0.86	0.91	0.88	33
**non-opening**	0.98	0.99	0.99	760
**accuracy**			0.98	873
**macro avg**	0.93	0.92	0.92	873
**weighted avg**	0.98	0.98	0.98	873

## Data Availability

Data is contained within the article.
